# What You Need to Know Before Implementing a Clinical Research Data Warehouse: Comparative Review of Integrated Data Repositories in Health Care Institutions

**DOI:** 10.2196/17687

**Published:** 2020-08-27

**Authors:** Kristina K Gagalova, M Angelica Leon Elizalde, Elodie Portales-Casamar, Matthias Görges

**Affiliations:** 1 Canada’s Michael Smith Genome Sciences Centre BC Cancer Vancouver, BC Canada; 2 Bioinformatics Graduate Program University of British Columbia Vancouver, BC Canada; 3 Research Institute BC Children’s Hospital Vancouver, BC Canada; 4 School of Population and Public Health University of British Columbia Vancouver, BC Canada; 5 Department of Pediatrics University of British Columbia Vancouver, BC Canada; 6 Department of Anesthesiology, Pharmacology and Therapeutics University of British Columbia Vancouver, BC Canada

**Keywords:** database, data warehousing, data aggregation, information storage and retrieval, data analytics, health informatics

## Abstract

**Background:**

Integrated data repositories (IDRs), also referred to as clinical data warehouses, are platforms used for the integration of several data sources through specialized analytical tools that facilitate data processing and analysis. IDRs offer several opportunities for clinical data reuse, and the number of institutions implementing an IDR has grown steadily in the past decade.

**Objective:**

The architectural choices of major IDRs are highly diverse and determining their differences can be overwhelming. This review aims to explore the underlying models and common features of IDRs, provide a high-level overview for those entering the field, and propose a set of guiding principles for small- to medium-sized health institutions embarking on IDR implementation.

**Methods:**

We reviewed manuscripts published in peer-reviewed scientific literature between 2008 and 2020, and selected those that specifically describe IDR architectures. Of 255 shortlisted articles, we found 34 articles describing 29 different architectures. The different IDRs were analyzed for common features and classified according to their data processing and integration solution choices.

**Results:**

Despite common trends in the selection of standard terminologies and data models, the IDRs examined showed heterogeneity in the underlying architecture design. We identified 4 common architecture models that use different approaches for data processing and integration. These different approaches were driven by a variety of features such as data sources, whether the IDR was for a single institution or a collaborative project, the intended primary data user, and purpose (research-only or including clinical or operational decision making).

**Conclusions:**

IDR implementations are diverse and complex undertakings, which benefit from being preceded by an evaluation of requirements and definition of scope in the early planning stage. Factors such as data source diversity and intended users of the IDR influence data flow and synchronization, both of which are crucial factors in IDR architecture planning.

## Introduction

### Background

An electronic health record (EHR) is a system for the input, processing, storage, and retrieval of digital health data. EHR systems have been increasingly adopted in the United States over the past 10 years [1], and their use is spreading worldwide in both hospital and outpatient care settings [2,3]. An EHR is typically organized in a patient-centric manner and has become a powerful tool to store data in a time-dependent and longitudinal structure. EHR data can also be integrated into an enterprise data warehouse or integrated data repository (IDR). IDRs collect heterogeneous data from multiple sources and present them to the user through a comprehensive view [4]. Unlike EHRs, IDRs offer specialized analytical tools for researchers or analysts to perform data analyses.

An IDR is a significant institutional investment in terms of both initial costs and maintenance, but it offers the advantage of clinical data reuse beyond direct clinical care, such as for research and quality improvement studies. Secondary use of clinical data is a rapidly growing field [5,6]; an increasing number of institutions have implemented in-house IDRs and several others are developing IDRs for future research endeavors.

Unlike clinical practice, which focuses on enhancing the well-being of current patients, the purpose of an IDR is to produce generalized knowledge that can be extended to future patients. Typical applications of IDRs include retrospective analysis and hypothesis generation [7]. Some IDRs also support clinical applications, such as clinical decision support systems (CDSSs), that work alongside clinical practice to estimate risk factors or predictive scores associated with clinical treatments. CDSSs help to avoid medical errors and deliver efficient and safer care by assisting the provider with diagnosis, therapy planning, and treatment evaluation decisions [8]. All these applications are valuable resources that have the potential to improve the quality of health care [9] and reduce health costs if implemented appropriately [10].

### Objective

Our study is motivated by the need to develop a pediatric IDR at our institution and by the lack of literature providing practical recommendations to apply during the initial development stages. Reviews by Shin et al [11] and Huser et al [12] highlighted the recommended characteristics when designing an IDR; however, they include only a small set of examples and a limited number of example IDRs. Since 2014, the IDR landscape has evolved rapidly, and thus, we felt more recent developments needed to be better addressed as well. A 2018 review by Hamoud et al [13] provided a comprehensive description of most recent data warehouses, including information about their data content, processing, and main purpose; it also provides general recommendations for the implementation of an IDR, but no practical considerations to guide the planning stages.

This study compares the features of contemporary IDRs and presents some guiding principles for the design and implementation of a clinical research data warehouse. Our research objective was to identify the major features of contemporary IDRs and obtain a list of established architectures used in the field of health informatics. We expect that this review will be useful for other small- to medium-sized institutions that plan to implement an institutional IDR and have no extensive experience in the field.

## Methods

We conducted a literature review and a targeted web-based search to identify the major existing IDRs and synthesized the retrieved information around key themes.

### Literature Review Search

**Figure 1:**
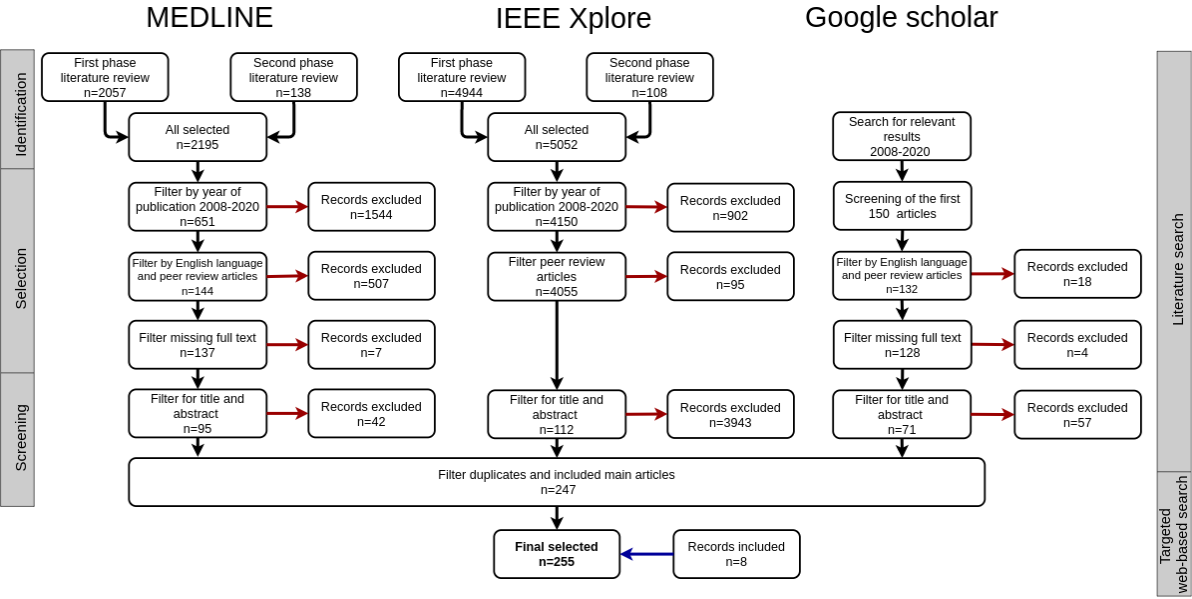
Article selection process. The diagram shows the number of articles at each stage of selection for each of the 3 databases: MEDLINE (Medical Literature Analysis and Retrieval System Online), IEEE Xplore (Institute of Electrical and Electronics Engineers Xplore), and Google Scholar.

We performed a narrative review following the procedure described below. First, a literature search was conducted using Ovid MEDLINE (Medical Literature Analysis and Retrieval System Online) and IEEE Xplore (Institute of Electrical and Electronics Engineers Xplore), queried in March 2020 (Figure 1). Articles were identified in 2 iterative phases. The first phase used an initial list of keywords querying for infrastructure purposes (data integration, such as linkage and harmonization) as well as infrastructure type and hospital setting (Multimedia Appendix 1: A1). The second phase search used additional keywords identified from the titles and abstracts of articles retrieved in the first phase (Multimedia Appendix 1: A1). Second, Google Scholar was queried for major article keywords (Integrated Data Repository) OR (Clinical Data Warehouse), and the first 150 retrieved hits were screened. The query was executed in a single search stage because the traditional search methods using Ovid MEDLINE and IEEE Xplore already produced exhaustive results.

We selected peer-reviewed articles, published in the English language between January 2008 and March 2020, to include the most current data warehouse features. Non-English articles were excluded because of a lack of resources for translation. We retained articles for which the full text was available and removed duplicates. KG read the abstracts, and the articles describing specific data integration strategies, describing architecture structures, or providing more information about the data models were included. When it was unclear whether an article should be included, the authors EPC and MG were consulted. Duplicated articles were removed using EndNote reference management software (Clarivate Analytics). Additional articles providing the most up-to-date information about selected IDRs or cited by the selected articles were included in the selection process because they were considered relevant for the IDR definition. Targeted Web-Based Search of Known Institutional IDRs

We manually queried nonpublished resources with the goal of adding contemporary data warehousing practices implemented in large North American hospitals. A convenience sample of hospitals known to be leaders in these types of data warehousing was suggested by EPC and MG.

Additionally, we browsed publicly available information on each of the targeted institutional websites (Multimedia Appendix 1: A2). This was complemented with relevant peer-reviewed articles cited in these websites related to the design, implementation, and applications of such repositories.

### Manual Shortlisting for a Comparative Review Analysis

For the comparative review analysis, we performed a manual selection to shortlist articles specifically describing IDR architectures. The shortlisting considered the major focus of the article and the presence of significant details describing data integration, data processing, or database services. The selected articles were searched for related IDR projects and further web-based resources (Table 1 and Multimedia Appendix 1: A3).

**Table 1:** Institutions and major features of the integrated data repositories.

IDR^a,b^		IDR scope	Architecture model	Standard common data model	Standard terminologies	Primary references
**The National Institutes of Health Clinical Center-Small-sized institution**						
	Biomedical Translational Research Information System (BTRIS)	General care	General	N/A^c^	RED^d^	[14]
	Deceased subjects (dsIDR)	Deceased subjects	General	N/A	RED	[15]
**University of Kansas Medical Cent** **er-Medium-sized institution**						
	Healthcare Enterprise Repository for Ontological Narration (HERON)	General care	General	i2b2^e^	ICD^f^-9/ICD-10, CPT^g^, RxNorm^h^, SNOMED-CT^i^, NDFRT^j^, NCI^k^, FDB^l^	[16,17]
**Stanford University Medical Center-** **Medium-sized institution**						
	Stanford Translational Research Integrated Database Environment (STRIDE)	General care	General	i2b2, OMOP^m^	ICD-9, CPT, RxNorm, SNOMED-CT	[18,19]
	STAnford Research Repository (STARR)	General care	General	i2b2, OMOP	ICD-9, CPT, RxNorm, SNOMED-CT	[20]
**The Georges Pompidou University Hospital (HEGP)-Medium-sized institution**						
	HEGP CDW^n^ platform	General care, cardiovascular, cancer	General	i2b2	ICD-10, LOINC^o^, SNOMED-CT	[21-23]
**Hanover Peter L. Reichertz Institute-** **Large-sized i** **nstitution**						
	Hanover Medical School Translational Research framework (HaMSTR)	General care	General	i2b2	ICD-10, LOINC	[24]
**Erlangen University Hospital-Large-sized institution**						
	Clinical data warehouse	General care	General	I2b2	LOINC, NCI	[25]
**Seoul St. Mary Hospital-Large-sized institution**						
	Prostate cancer research database	Cancer	General	N/A	N/A	[26]
**Lead partner: Cincinnati Children’s Hospital Medical Center-Collaborative project**						
	Maternal and Infant Data Hub (MIDH)	Perinatal	General	OMOP	ICD-9/ICD-10, SNOMED-CT	[27]
**Georges Pompidou, Cochin and Necker Hospitals-** **Collaborative project**						
	CAncer Research for PErsonalized Medicine (CARPEM)	Cancer	General	Variant of i2b2 (tranSMART^p^)	ICD-9/ICD-10, SNOMED-CT, ATC^q^, GO^r^, HPO^s^	[28]
**Learning Healthcare System (LHS) across South Carolina-Collaborative project**						
	Health Science South Carolina (HSSC) clinical data warehouse	General care	General	i2b2	N/A	[29]
**Windber Research Institute-Collaborative project**						
	Data Warehouse for Translational Research (DW4TR)	Cancer	General	N/A	MeSH^t^, SNOMED-CT, NCI, caBIG VCDE^u^	[30,31]
**Veterans’ Health Administration-Collaborative project**						
	VA EHR (Veterans Administration’s electronic health records)	General care	General with CDSS	N/A	ICD-9	[32]
**Coordinated by Medtronic Iberica SA-** **Collaborative project**						
	Models and simulation techniques for discovering diabetes influence factors (MOSAIC)	Diabetes	General with CDSS	i2b2	ICD-9, DRG^v^, ATC	[33]
**National collaboration-** **Collaborative project**						
	China Stroke Data Center (CSDC)	Cerebrovascular	General with CDSS	N/A	N/A	[34]
**Houston Methodist Hospital-Large-sized institution**						
	Methodist Environment for Translational Enhancement and Outcomes Research (METEOR)	General care	General with CDSS	Extension of i2b2	ICD-9, CPT	[35]
**Mayo Clinic-** **Large-sized institution**						
	Mayo Enterprise Data Trust (MEDT)	General care	General	i2b2	LexGrid^w^	[36]
	Ovarian cancer registry	Cancer	General	i2b2	LexGrid	[37]
	Translational Research Center (TRC)	Cancer	Biobank-driven	i2b2	LexGrid	[38]
**Vanderbilt University Medical Center-** **Large-sized institution**						
	Synthetic Derivative	General care	General	N/A	FDB, ICD-9, CPT	[39]
	BioVU	General care	Biobank-driven	N/A	FDB, ICD-9, CPT	[40]
**The Children's Hospital of Philadelphia-** **Medium-sized institution**						
	Biorepository Portal (BRP)	Cancer, pediatric	Biobank-driven	Harvest	N/A	[41]
**University of São Paulo-Large-sized institution**						
	BioBankWarden (BBW)	Cancer	Biobank-driven	N/A	ICD-10, SNOMED-CT, LOINC, GO	[42]
**University of Pavia and Fondazione S. Maugeri-** **Large-sized institution**						
	onco-i2b2	Cancer	Biobank-driven	i2b2	SNOMED-CT	[43]
**Leon Berard Cancer Center-** **Small-sized institution**						
	CLB-IT^x^	Cancer	User-controlled application layer	N/A	ADICAP^y^, ICD-O	[44]
**Lead partner: University of Utah-Collaborative project**						
	Federated Utah Research and Translational Health electronic Repository (FURTHeR)	Several	Federated	i2b2, OMOP, OpenMRS^z^	ICD-9/ICD-10, LOINC, SNOMED-CT, RxNorm	[45]
	OpenFurther	Several	Federated	i2b2, OMOP, OpenMRS	ICD-9/ICD-10, LOINC, SNOMED-CT, RxNorm	[46]
**Lead partner: University of Utah-Collaborative project**						
	Pediatric Health Information System (PHIS+)	Pediatric	Based on FURTHeR	i2b2	LOINC, SNOMED-CT	[47]
**@neurIST** **European Project-Collaborative project**						
	@neurIST platform	Cerebrovascular	Federated as in FURTHeR	N/A	@neurIST ontology^aa^	[48]
**University Clinics in Northern Germany-** **Collaborative project**						
	Research Data Management System (RDMS)	Cancer	Federated as in FURTHeR	i2b2	ICD-10, SNOMED-CT	[49]

^a^IDR: Integrated Data Repository

^b^The IDRs are defined by their data scope, architecture model (as defined by the major design class represented in Figure 2), standard common data model, standard terminology, and primary reference.

^c^N/A: not applicable, n=4 in Standard Terminology.

^d^RED: Research Entities Dictionary, n=1.

^e^i2b2: Informatics for Integrating Biology and the Bedside

^f^ICD-9/ICD-10/ICD-O: International Classification for Diseases, version 9/10, O for oncology, n=14.

^g^CPT: Current Procedural Terminology, n=4.

^h^RxNorm: standardized nomenclature for clinical drugs, n=3.

^i^SNOMED-CT: Systematized Nomenclature of Medicine-clinical terms, n=11.

^j^NDFRT: National Drug File Reference Terminology, n=1.

^k^NCI: National Cancer Institute, n=2.

^l^FDB: First Databank, n=2.

^m^OMOP: Observational Medical Outcomes Partnership

^n^HEGP CDW: Hôpital Européen Georges Pompidou Clinical Data Warehouse, n=1.

^o^LOINC: Logical Observation Identifiers Names and Codes, n=5.

^p^tranSMART: Open-source data platform for translational research, n=1.

^q^ATC: Anatomical Therapeutic Chemical Classification, n=2.

^r^GO: Gene Ontology, n=2.

^s^HPO: Human Phenotype Ontology, n=1.

^t^MeSH: Medical Subject Headings; n=1.

^u^caBIG VCDE: the cancer Biomedical Informatics Grid Vocabulary and Data Elements Workspace, n=1.

^v^DRG: Diagnosis Related Group, n=1.

^w^LexGrid: Lexical Grid, n=1.

^x^CLB-IT: Léon Bérard Cancer Center-IT.

^y^ADICAP: Association pour le Développement de l'Informatique en Cytologie et en Anatomie Pathologique, n=1.

^z^OpenMRS: Open Medical Record System, n=1.

^aa^@neurIST ontology, n=1.

**Figure 2:**
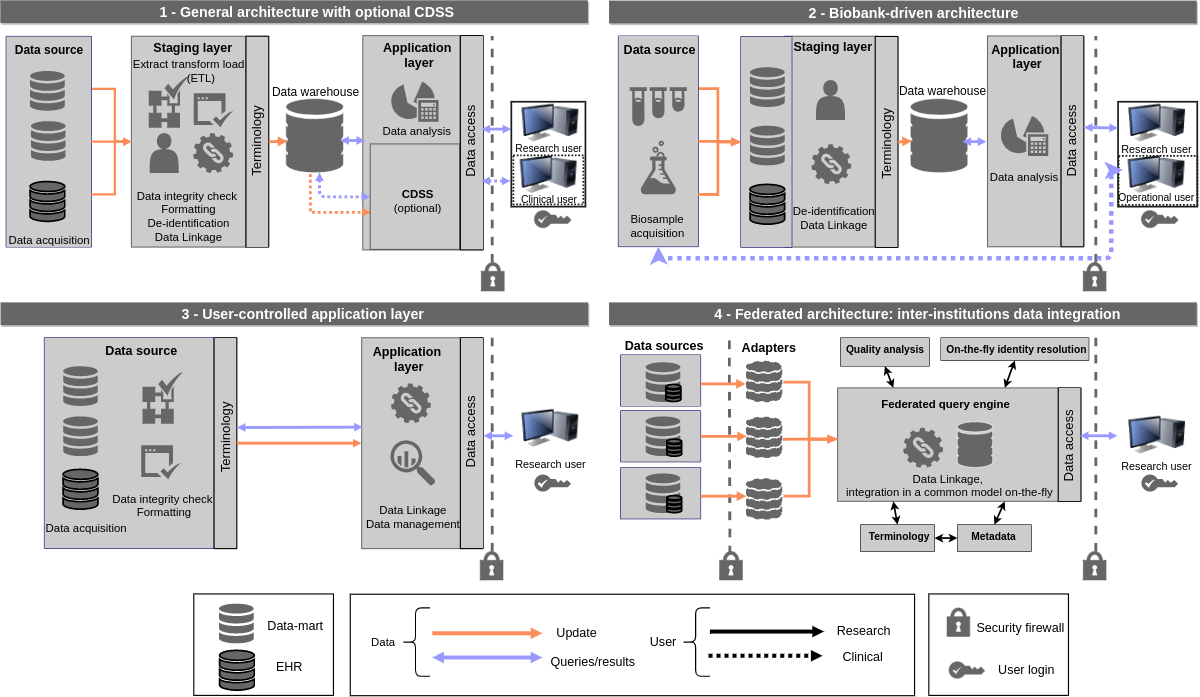
Architecture models identified from selected integrated data repositories (IDRs). Arrows indicate data output because of a query (blue) and data input (orange) because of data integration or update. Continuous lines show data query and integration applied by research users, whereas dashed lines are data queries performed by operational or clinical users.

### Literature Synthesis and Institution Characterization

Information from the literature was aggregated through thematic analysis and collapsed into 4 classes of IDR architectures. We evaluated the main features of the identified IDRs, such as data processing components, data characteristics, common terminologies, and data models. Features were summarized, compared, and contrasted. We extracted information about host institutions and divided them into small (≤500 beds), medium (500-1000 beds), and large (>1000 beds) institutions based on the number of beds listed on the institution’s websites.

### Analysis of Word Content

Selected articles were uploaded into NVivo 12 (QSR International LLC) for qualitative analysis, specifically to count the word frequency in the selected papers. The words with a minimum length of 5 in the full text were counted, excluding stop words, and grouped by synonyms. The word frequency is represented as a word cloud, generated with R (R Foundation for Statistical Computing) and *wordcloud* package 2.6.

### Citations Analysis

The references of the articles describing IDRs were downloaded in a semiautomated manner using Content Extractor and Miner software [50] to parse the full-text PDF files. References to web resources, video-cast meetings, and software were removed, and partial references were manually corrected. The references were grouped by *first author* and *year of publication* and loaded in R (R Foundation for Statistical Computing) and plotted with UpSetR [51].

## Results

### Overview

A total of 241 articles were identified in the literature search [11,13-19,21-29,31,33-35,37,43,44,47-49,52-264]; the largest number of articles were identified in IEEE Xplore (n=112), followed by MEDLINE (n=95), and Google Scholar (n=71). After removing duplicates (n=24), we added 3 articles that were frequently cited in the selected articles but were missing from our search results [30,36,42]. Three articles [38,40,45] were further added that provided additional details relevant to the review topic. Finally, 1 article was replaced by a more updated publication [265]. These 247 articles were combined with the targeted web-based search [32,39-41,266-269]; hence, we identified a total of 255 articles (Figure 1). The most frequent words in the articles were *system,* *information*, *study*, *project*, and *design* (Multimedia Appendix 1: A4.1). A total of 79 of these 255 articles were published between 2014 and 2016, and 34 were published in 2019; this date range covers the full range of initially identified articles in this domain area (Multimedia Appendix 1: A4.2 and A4.3).

A total of 116 articles were presented in proceedings of international scientific conferences, particularly those published in the book series Studies in Health Technology and Informatics (n=23); this included the World Congress of Medical and Health Informatics and Medical Informatics Europe. The second most frequent proceedings were the American Medical Informatics Association annual symposium and joint summits on translational science (n=12). The most frequently observed journals were the Journal of the American Medical Informatics Association (n=9) and BioMed Central (BMC; n=8), with BMC Bioinformatics being the most common. More details about the individual conferences and journals can be found in Multimedia Appendix 1: A4.4 and A4.5.

For this review, we focused on the 34 articles describing 29 IDRs for which sufficient design details were presented. The additional web resources describing 2 IDRs, Stanford Translational Research Integrated Database Environment (STRIDE) and Federated Utah Research and Translational Health Electronic Repository (FURTHeR), referred to novel projects STAnford Research Repository (STARR) [20] and OpenFurther [46], respectively, which increased the number of IDRs to 31 from 25 different institutions or collaborative projects (Table 1). In reviewing the references in these 34 articles, we observed only a small overlap, with 1 reference [270] being found in common in a maximum of 11 articles (Multimedia Appendix 1: A5.1). The most frequently cited among the 34 are onco-Informatics for Integrating Biology and the Bedside (i2b2) [43,271], STRIDE [18], and the Mayo Clinic [36] IDRs, cited in 8, 5, and 4 articles, respectively (Multimedia Appendix 1: A5.2).

IDRs represent a variety of applications of health data warehousing for research. Although they share common characteristics, as described in detail below, they also demonstrate the many different purposes they can serve. For example, BioVU [40] and the Synthetic Derivative [39] at Vanderbilt University Medical Center are examples of a biobank-driven database that automatically couples patients’ clinical information to biological samples (biosamples). The power of this system is its connection between genotype and phenotype and its large number of biosamples (>50,000), which allows a rich set of cohort research studies. The Maternal and Infant Data Hub (MIDH) at Cincinnati Children’s Hospital Medical Center [27] is a regional perinatal data repository that integrates a large and diverse set of data from different institutions. The strength of the project is the combination of delivery and postdischarge hospital data and the linked mother and child data sets. The pilot database contains approximately 70,000 newborns and 42,000 pediatric postnatal visits. Another example is the Hanover Medical School Translational Research Framework (HaMSTR) framework at the Hanover Peter L. Reichertz Institute [24], which was developed to automatically load data from a clinical data repository into a standard data model that researchers can query; it is a successful example of fast data upload and query using data structures designed from standard data models available for clinical research.

### Characteristics of the Institutions in the Selected IDR Sample

We identified 2 types of IDRs: those developed for use in a single institution (n=19) and those implemented for a collaborative project (n=12). The latter typically integrate patient data and provide project-specific tools. The median number of different institutional partners in a collaborative IDR is 6, with one of the partners acting as an organizational hub. The partners range from research institutes, laboratories, and private institutions to university medical centers.

The IDRs were further divided by their scope (Table 1), which were classified as general or specialized medical care (cancer, pediatrics, perinatal, cerebrovascular, or cardiovascular). Seven of the 10 IDRs containing specialized data were collaborative projects, likely indicating the need to pool data from several institutions when dealing with smaller but more focused patient populations.

### Four Major Architecture Models Used in Our Selected IDR Sample

We identified 4 overarching conceptual architectures that summarize the data layers in the selected IDRs (Figure 2). Different institutions can implement multiple architectures for different purposes; we assigned each IDR to a category considering the major features of the IDR, as described in their respective articles.

The general architecture model is the most common model, with 19 identified IDRs structured around medical data mining (Figure 2, *General architecture with optional CDSS*). In outline, different data marts are transferred to a staging layer that harmonizes the input to a common data view; data are loaded into a common data warehouse and queried through an application layer that communicates with the user; a CDSS tool can provide added functionality. Hence, in this architecture, each data source is originally stored in an independent data mart, collecting data from a separate research or clinical source within the same institution. Data are processed in the staging layer, which reshapes the input to an integrated view through several steps of data linkage, transformation, and harmonization. The next stage of processing is loading the data into a single database connected to an application layer that provides the tools for end users, typically researchers, to access and analyze the data securely with different services. An example of an IDR providing multiple services is the STRIDE architecture stack [18], which includes several services for data analysis or research data management. The articles describing METEOR [35], CSDC [34], models and simulation techniques for discovering diabetes-related factors (MOSAIC) [33], and Veterans Administration’s EHRs [32] provide further details about the integration of CDSS tools in the architecture. In these cases, the architecture model is divided into CDSS and data analysis modules, both of which communicate with the common database. The CDSS allows clinical staff to retrieve real-time individual patient records and to use analytical models to make risk prediction. The CDSS tools described by METEOR and MOSAIC, for example, learn from the clinical data stored in the data warehouse and estimate risk factors predicting hospital readmission or long-term complications.

The Health Science South Carolina (HSSC) [29] IDR gathers data from different clinical systems implemented in various institutions, all of which are party to a data collaboration agreement that authorizes data aggregation in a single data warehouse. This data warehouse contains a longitudinal record for each individual across all institutions. Data processing and terminology mapping occur in a conceptual staging layer, as in the case of the general architecture model.

In the case of the Erlanger University Hospital IDR [25], terminology is mapped using vocabularies that are manually curated and mapped through an automatic workflow that processes the raw data to the final data warehouse format. Other IDRs that make use of multiple terminologies are health care enterprise repository for ontological narration [16], Research for PErsonalized Medicine (CARPEM) [28], and STRIDE [18], but further details of their mapping processes were not available.

The biobank-driven architecture model is built around a particular application, in this case, biobanking (Figure 2, *Biobank-driven architecture*). This model is similar to the general architecture model but, in this case, the IDR is built around the biosamples database. The biosample data integration occurs at the staging layer. The main feature is that the model allows the biosample operational user to access the raw and identified biobank data source for quality control and biosample management. An example of a biobank-driven structure is the biorepository portal (BRP) [41,266], which allows for the automatic integration of biosamples with clinical data, while maintaining unrestricted access to the biorepository for the operational team. The Mayo Clinic and Vanderbilt University adopt the general and biobank-driven architecture models in parallel.

The user-controlled application layer architecture model does not have a specific staging layer (Figure 2, *User-controlled application layer*). This architecture does not include a central data warehouse; the data are preprocessed and integrated from the original data sources only when the users query the data. Hence, data are processed in 2 stages: the first stage preprocesses the original data to a common format. The user query then carries out the final data integration function for the output delivery. In this architecture, a common data warehouse is not implemented, but rather the data are dynamically queried. An example is the text mining technology at the Léon Bérard Cancer Center (CLB) [44], which indexes text documents during the preprocessing stage and in which the users’ queries return the exact documents matched.

The federated architecture is implemented for heterogeneous data retrieval and integration across multiple institutions (Figure 2, *Federated architecture*, adapted from OpenFurther). In this case, institutions selectively share their data through an adaptor system that applies common preprocessing, with data integrated on-the-fly in a virtual data warehouse. The FURTHeR federated query platform [45] builds a virtual IDR that responds to the needs of the user and calls several services for data resolution on-the-fly and upon query. The architecture model is flexible and operates using several services for data integration. An application of FURTHeR is the Pediatric Health Information System+ project [47], which combines data from 6 institutions. The IDR uses a federation component, which aggregates and stores translated query results in a temporary, in-memory database for presentation and analysis by the researcher for the duration of the user’s session. Federated data integration was also proposed using a research data management system (RDMS) [49], which integrates clinical and biosample data from several institutions in Germany. The @neurIST [48] is a large IDR dedicated to translational research that includes data, computing resources, and tools for researchers and clinicians. Data are located across different sites and are securely shared with a grid infrastructure that allows federated data access.

The 4 types of architecture present different analytics tools, data presentation logic, and query interface based on the type of user they serve, which can be classified into 2 major groups: the first group, such as researchers and operational or business analysts, uses the IDR to identify important clinical features that occur at the level of patient cohorts. The second type of user, such as physicians and other health care professionals, uses the IDR to make decisions at an individual patient level, for example, to plan specific therapeutic interventions or predict risk. The first type of user is served by all the architecture models (*Research user* in Figure 2). The general architecture model that incorporates a CDSS presents a clear separation of both user types who have different applications for IDR data, with CDSS queries being made by clinical users (Figure 2, *General architecture with optional CDSS*). Similarly, the biobank-driven architecture model includes operational users who can directly query the information regarding patient biosamples for clinical applications (Figure 2, *Biobank-driven architecture*).

### Data Retrieval and Update Are Influenced by the IDR Architecture Model

Both data update and integration schedules in an IDR are important features that define the timeliness of data. Here, we describe some of the key limiting steps and their occurrence in the different IDR architecture models.

#### Data Retrieval

The data processing involved in extraction, transformation, and loading (ETL) is described in detail in the articles of biomedical translational research information system (BTRIS) [14], HaMSTR [24], Mayo Translational Research Center (TRC) [38], CARPEM [28], onco-i2b2, Vanderbilt’s Synthetic Derivative [39] and BioVU [40], and BRP [41]. These IDRs represent the general and biobank-driven architecture models, which implement a staging layer for the ETL process. A temporal sequence of the ETL steps is as follows:

1. Data extraction from source(s): The source data are extracted by an automatic (or manual) process.
2. Deidentification: Identifiable patient features, such as demographics or localization, are removed before loading into the IDR. The biobank-driven IDRs implement an automated process of this step without the need for extensive institutional reviews. In addition to the deidentified data, BTRIS [14] and Vanderbilt’s Synthetic Derivative [39] maintain a parallel database with original identifiable patient entries for research purposes where appropriate.
3. Assignment of unique identifiers: Deidentified data are assigned unique patient identifiers that are used as a reference for linking.
4. Data transformation and standardization: Data are first checked for possible errors or missing values and are then transformed into a common format that is standard for all cohorts. Data may be subjected to transformation, such as the derivation of new values from the existing ones (pseudonymization) for maintaining privacy.
5. Standard terminology and ontology mapping: Data types are labeled with standard terminologies.
6. Data linkage: If the data are derived from multiple sources, they are linked and combined in the IDR.
7. Loading into the data warehouse: This is performed by either an update of existing data or a complete data re-import into the data warehouse.

The CLB [44] IDR (user-controlled application layer architecture model) uses specialized software to manipulate the content from unstructured data without using an ETL process. IDRs representing architecture model 4 do not provide additional information on the ETL process in their respective articles.

#### Data Update

Five of the selected articles provide additional information about the frequency of data updates in their IDRs. BTRIS [14] and Vanderbilt’s Synthetic Derivative [39] argue for daily IDR updates as new source data accumulate daily. Onco-i2b2 [43] performs more frequent data synchronization, as frequent as every 15 min. A real-time data update is presented by METEOR [35] and MOSAIC [33], which also integrate a CDSS in their architecture model and thus need this frequency to make actionable decisions. MOSAIC presents an example with asynchronous data update; although the CDSS is updated in real time, the demographics are synchronized only every 6 months. The general architecture model combined with a CDSS may require real-time data updates, whereas the general or the biobank-driven architecture models, without a CDSS, may have periodic updates that vary widely in frequency.

### Major IDR Features: Data Type, Standard Terminology, and Common Data Model

#### Data Type

**Figure 3:**
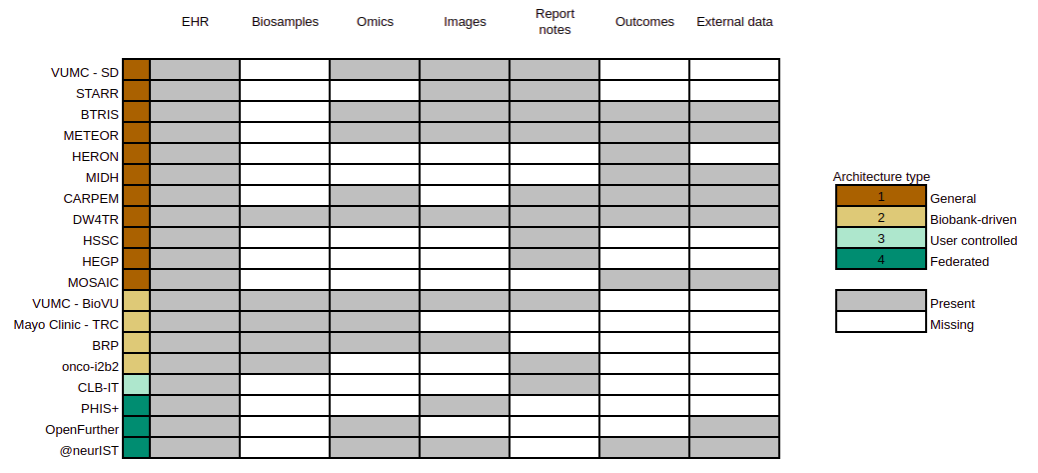
Common data types across IDRs. Columns show the main types of data collected in the selected IDRs. Gray-filled cells denote feature presence, with colors classifying the IDRs based on the examined architectures. Only 19 IDR articles contained enough information in their articles to be included in this figure. BRP: biorepository portal; BTRIS: biomedical translational research information system; CARPEM: cancer research for personalized medicine; CLB-IT: Léon Bérard Cancer Center Information Technology; DW4TR: Data Warehouse for Translational Research; EHR: electronic health record; HEGP: Hôpital Européen Georges Pompidou; HERON: health care enterprise repository for ontological narration; HSSC: Health Science, South Carolina; IDRs: integrated data repositories; Mayo Clinic-TRC: Mayo Clinic – Translational Research Center; METEOR: Methodist Environment for Translational Enhancement and Outcome Research; MIDH: Maternal and Infant Data Hub; MOSAIC: models and simulation techniques for discovering diabetes-related factors; Onco-i2b2; PHIS+: Pediatric Health Information System+; STARR: STAnford Research Repository; VUMC-BioVU: Vanderbilt University Medical Center–BioVU; VUMC-SD: Vanderbilt University Medical Center–Synthetic Derivative.

We have listed the data types in 19 of the selected IDRs based on information in the articles (Figure 3). The most common types of data are those extracted from EHR that include patient demographics, diagnoses, procedures, laboratory tests, and medications.

Several IDRs incorporate data from biosamples and their omics characterization, especially those based on the biobank-driven architecture model such as TRC [38], BRP [41,266], and BioVU [40]. Other examples of omics-based IDRs are CARPEM [28], Data Warehouse for Translational Research (DW4TR) [30], and @neurIST [48], which are dedicated to specific domains of research, namely cancer and cerebral aneurysm research.

Several types of images are part of modern IDRs, such as radiographic images in BTRIS [14] and document images in Methodist Environment for Translational Enhancement and Outcome Research (METEOR) [35]. In addition, medical reports are integrated in the IDRs. Clinical documents can be processed using natural language processing (NLP) algorithms to extract clinical conditions, medication types, and other features from common hospital procedures, which increases their utility through transformation into structured data. NLP modules are integrated in CLB-IT [44], which is specifically built for text processing entries, as well as BTRIS [14], METEOR [35], and onco-i2b2 [43].

IDRs including CDSS include outcome data types, which are relevant for calculating risk factors or predictive values in clinical domains. External data can also be integrated into the IDRs, including genomics data from disease model organisms (BTRIS) [14], patients from external sources (BTRIS [14] and DW4TR [30]), or environmental indices and geolocation (MIDH) [27].

#### Standard Terminology

Health information technology uses controlled terminologies to condense the information to a set of codes that can be manipulated more easily and automatically in data processing. We observed the adoption of both common [272,273] and specialized terminologies (eg, Anatomical Therapeutic Chemical Classification [274], human phenotype ontology [275], Gene Ontology [276]). The most broadly used were International Classification of Diseases (ICD)-9 and 10 for the classification of diseases, systematized nomenclature of medicine-clinical terms (SNOMED-CT) for a variety of medical domains, Logical Observation Identifiers, Names, and Codes for laboratory observations, and current procedural terminology for common procedures (Table 1). These terminologies were utilized within the EHR and further integrated into the IDRs.

#### Common Data Model

A common data model (CDM) is a standard data schema that enables data interoperability and sharing. Contemporary data warehouses propose an analytical platform built around the CDM that provides all the software components to construct and manage the data in a CDM. A few different CDMs have been developed and adopted by the wider clinical research community, although some institutions still favor using a custom data schema tailored to their specific needs. In our study, a standard CDM was adopted by 18 of the 29 IDRs. The most frequently applied CDM, found in 16 instances, is Informatics for Integrating Biology and the Bedside (i2b2) [277]. METEOR [35] applies i2b2 with an expanded schema, and CARPEM [28] applies tranSMART [278], which is a framework layered on top of i2b2, dedicated to integrating omics data with EHR data. Another popular CDM that has been used more frequently in recent years is the Observational Medical Outcomes Partnership (OMOP) [279], adopted by 3 IDRs, namely MIDH [27], OpenFurther [46], and STARR [20]. OpenFurther uses OpenMRS [280], which is an open-source software and CDM that delivers health care in low- and middle-income countries. The BRP [41] is the only example using Harvest as their CDM.

## Discussion

### Principal Findings

Our review identified several institutions of various sizes and scopes that utilize an IDR. These IDRs contain data used for both research and clinical decision-making purposes. The use of structured data from natural language processing of clinical notes, clinical imaging, and omics data are the most recent big data types to be integrated with standard clinical observations. Owing to the large heterogeneity, however, integration is complex and tailored to the specific needs during the IDR implementation and maintenance, as ETL necessitates a significant effort in both the initial modeling and the ongoing updates.

As a novel contribution, we proposed and classified IDR architectures into 4 major models that highlight the processing and integration steps. The most common architecture model employs a staging layer implemented before the data are loaded into the data warehouse.

A set of common features are applied across most IDRs: IDRs commonly use standard terminologies such as ICD-9/10 and SNOMED-CT, which are often already part of the EHR data. Several IDRs use an open-source translational research framework to model their data, as described by Huser et al [12]. We observed extensive use of i2b2 CDM and the emergent adoption of OMOP CDM, which has the possibility to map additional domain-specific terminologies. Interestingly, PCORnet is one of the newest CDMs, but its application was not discussed in the sample of IDRs reviewed. The PCORnet is the most recently implemented CDM that borrows from several other CDMs and is organized around patient outcomes [261].

To safeguard the data in the IDR, data security and privacy need to be ensured from the initial steps of development. Data security is an important factor in all architecture types, with a particular need in collaborative projects that share data across jurisdictions. For example, in the general architecture of HSSC [29], data need to be stored in physically and logically secure facilities, where data management is extended to all the parties involved, and data need to be transmitted between the participating institutions through private high-speed networks. In the case of federated data warehouses, such as @neurIST [48], there is a tight control of data flow between different institutions and clinical and research domains, following policies aligned with recommendations from the Legal and Ethics Advisory Board. Privacy, referring to the protection of patient’s personal information, emerged as an important feature, especially in the biobank-driven architecture; here, identifiable patient information is deleted from both the biosamples and the patient clinical data. Developers at the Children’s Hospital of Philadelphia and the Children’s Brain Tumor Tissue Consortium created an electronic Honest Broker (eHB) and Biorepository Portal (BRP) eHB [41], which provides a method for patient privacy protection by removing all the exposure of the research staff to patient identifiers and automating the deidentification process. Following a different privacy-preservation approach, Vanderbilt’s Synthetic Derivative database [39] alters the patient data by obfuscating the true entries while preserving their time dependence.

### Guiding Principles

The implementation of an IDR is subject to several factors that must be considered before development. We identified 2 major factors: (1) the data stored in the IDR and (2) the scope of the IDR, either being exclusively used for research purposes or in combination with clinical or operational purposes, as shown in the general and biobank-driven architecture models. Data types, heterogeneity, and volume greatly influence system load, update, and query of the database. The scope of the IDR influences its primary end users, researchers, clinical users, or operational users, who have different needs and, thus, need access to different sets of tools to extract, analyze, and visualize the data. All the features influence both the data latency and the data synchronization, which are major elements in the model architecture. Moreover, available funding plays an important role in architecture decisions, as are considerations for future expansions.

Among the set of selected IDRs, we observed a number of collaborative projects that work within specialized medical domains, such as cancer or pediatrics. Collaborative IDRs are likely to integrate their data to increase the number of patients, thus increasing the statistical power of their respective cohorts.

On the basis of our analysis, we highlight the following guiding principles for small- to medium-sized institutions planning to implement an IDR:

1. The general architecture model, with or without CDSS, is the most straightforward to implement; the data staging layer facilitates ETL and data processing before loading into the data warehouse.
2. Select a standard CDM already in use by other institutions; both i2b2 and OMOP provide server and client services in a single unique platform that serves the user with all the necessary tools to set up a structured IDR.
3. Wherever possible, adopt standard terminologies; we listed the most common terminologies derived from the integration with EHR data (Table 1). One promising approach is that common terminologies are applied in the first phases of the IDR development with other, more specialized terminologies, added later as the project scope expands.
4. Finally, the data update requirements and ETL process design should be carefully considered, the level of automation, as these are the limiting stages in data integration and update.

Commercial electronic medical record platforms such as Epic, Cerner, Meditech, and Allscripts are dominant in large institutions. However, although some information about how to query underlying databases and application programming interfaces to communicate with these systems are available, little information on transforming such data into IDR is available in the literature, most likely because of their proprietary nature. Most vendors also sell tools for analysts to query and make use of data from these clinical production systems; however, they are not IDRs themselves and are not targeted toward secondary use for research.

As for lessons learned in the field, Epstein et al [281] demonstrate the feasibility of transferring the development of a perioperative data warehouse (schemas and processes) built on top of Epic’s database from one institution to another.

### Comparison With Prior Work

In their review, Hamoud et al [13] provided general requirements for building a successful clinical data warehouse, recommending a top-down approach to the initial stages of development. They recommended considering all the individual components of the final system to decrease integration obstacles when dealing with heterogeneous data sources.

Three major factors contributing to the success of IDRs were identified by Baghal [231] when developing their in-house IDR: (1) organizational, enhancing the collaboration between different departments and researchers; (2) behavioral, building new professional relationships through frequent meetings and communication between departments; and (3) technical improvements to deploy new self-service tools that empower researchers. Collectively, these factors increase the utility and adoption of IDRs in clinical research.

In addition, the report by Rizi and Roudsari [282] on lessons and barriers from their development of a public health data warehouse, which IDR developers might want to consider, specifically, not to underestimate technical challenges such as those related to extracting data from other systems, difficulties in modeling and mapping of data, as well as data security and privacy. Other considerations include leveraging the IDR to improve data quality at the source, implementing a data governance framework from the beginning, and ensuring that key organizational stakeholders endorse the project early and strongly [282].

### Limitations

Our search was not intended to be a systematic search; therefore, we may have missed some articles. An example of missing articles is those describing raw and unstructured data repositories, also referred to as *data lakes*, as these did not appear in our search results although we knew they exist. One of the *data lakes* was presented by Foran et al [207] as a file reservoir, integrated in the data warehouse schema. For researchers to access those data, it was necessary to use a *feeder* database before their upload to the final data warehouse.

Furthermore, we were able to report on the IDRs and IDR features described in the literature, possibly omitting smaller institutions that are not actively publishing in peer-reviewed journals. In an attempt to mitigate this issue, we searched the representative institutional websites to retrieve additional details about the IDR architectures. As shown in Multimedia Appendix 1 [283-288]: A2, several organizations provide further details about their architecture in GitHub repositories or institutional Wiki pages, which can be explored for additional information besides the published literature.

This review includes articles and web resources shortlisted according to aspects of the IDR architectures that were considered relevant. Providing an exhaustive coverage of all aspects of IDR implementation, such as tools designed to interact with the IDR, are better left for a dedicated review. An example of such tools is the *Green Button* project, which provides critical help in treating patients [289-292]. Examples of CDM-based tools, built around an application, are the @neurIST platform [48], @neurLink, and @neurFuse application suites that consist of research-oriented modules dedicated to knowledge discovery and image processing. CDSS tools such as Green Button, @neurIST applications, or many other existing frameworks are essential in providing sophisticated analyses to support clinicians, but are beyond the scope of our review.

### Conclusions

There is significant potential in the implementation of IDRs in health institutions, and their importance is evident from the growing number of projects developed in the past 10 years. Despite the common trends in IDR implementation observed in this study, there are also many variations. There are 2 major design factors, namely data heterogeneity and IDR scope, which need to be carefully considered before embarking on the IDR design and planning process.

Finally, we aim to apply the knowledge presented in this study for the implementation of a pediatric IDR at our institution. By sharing our experience of planning and designing our IDR with those joining the field or planning to implement an IDR for research purposes, we hope to contribute to future IDR endeavors.

## Appendix

Multimedia Appendix 1 Supplementary methods and results.

## Abbreviations

BMC: BioMed Central
BRP: biorepository portal
BTRIS: biomedical translational research information system
CARPEM: CAncer Research for PErsonalized Medicine
CDM: common data model
CDSS: clinical decision support system
CLB: Léon Bérard Cancer Center
DW4TR: Data Warehouse for Translational Research
EHR: electronic health record
ETL: extraction, transformation, and loading
FURTHeR: Federated Utah Research and Translational Health Electronic Repository
HaMSTR: Hanover Medical School Translational Research Framework
HSSC: Health Science, South Carolina
ICD: International Classification of Diseases
IDR: integrated data repository
IEEE Xplore: Institute of Electrical and Electronics Engineers Xplore
i2b2: Informatics for Integrating Biology and the Bedside
MEDLINE: Medical Literature Analysis and Retrieval System Online
METEOR: Methodist Environment for Translational Enhancement and Outcome Research
MIDH: Maternal and Infant Data Hub
MOSAIC: models and simulation techniques for discovering diabetes-related factors
NLP: natural language processing
OMOP: Observational Medical Outcomes Partnership
SNOMED-CT: systematized nomenclature of medicine-clinical terms
STARR: STAnford Research Repository
STRIDE: Stanford Translational Research Integrated Database Environment
TRC: Translational Research Center
