# Supplementary material for: What You Need to Know Before Implementing a Clinical Research Data Warehouse: Comparative Review of Integrated Data Repositories in Health Care Institutions
Source: JMIR Form Res. 2020 Aug 27;4(8):e17687. doi: 10.2196/17687 (PMC7484778; doi:10.2196/17687)
Supplement: Multimedia Appendix 1 [file formative_v4i8e17687_app1.pdf]

Multimedia Appendix

What You Need to Know Before Implementing a Clinical Research Data Warehouse: A Comparative Review of Integrated Data Repositories in Health Care Institutions

Kristina K. Gagalova, M. Angelica Leon Elizalde, Elodie Portales-Casamar, Matthias Görges

Table of contents

**A1** Literature keywords searches	2
**A2** Targeted web-based search of known institutional IDRs	4
**A3** Additional references consulted during the synthesis stage	5
**A4** Article summary statistics	8
**A5** Citations overlap of the main IDR articles	12

A1 Literature keywords search

FIRST PHASE - Medline

The initial query included the following concepts: 'Infrastructure Purpose' **AND** 'Infrastructure Type' **AND** 'Hospital Setting'. The selected terms embedded in the search command are shown in the highlighted areas.

Infrastructure Purpose

(data adj5 (integration or mining or link* or shar* or process*

Infrastructure Type

(data adj5 (hub or administrative or operational or repositor* or composite or cyberinfrastructure)).tw,kw. OR data biorepository OR cyberinfrastructure OR (biomedical adj5 (informatics or research)).tw,kw. OR Precision Medicine OR Information Systems OR Perioperative care

Hospital Setting

hospital*.tw,kw. OR hospitals OR hospitals, community OR hospitals, general OR hospitals, high-volume OR hospitals, low-volume OR exp hospitals, private OR exp hospitals, public OR hospitals, rural OR hospitals, satellite OR exp hospitals, special OR exp hospitals, teaching OR exp hospitals, urban OR mobile health units OR secondary care centers OR tertiary care centers

SECOND PHASE - Medline

The second query includes additional keywords retrieved from the first phase articles: 'Infrastructure Purpose' **AND** 'Infrastructure Type'. The selected terms embedded in the search command are shown in the highlighted areas.

Infrastructure Purpose

Personalized medicine OR translational research

Infrastructure Type

Information storage OR information retrieval OR information processing OR Database Management Systems OR electronic medical record system

FIRST PHASE – IEEE Xplore

The initial query included the following concepts: 'Infrastructure Purpose' **AND** 'Infrastructure Type' **AND** 'Hospital Setting', same as for the Medline search. The selected terms embedded in the search command are shown in the highlighted areas.

Infrastructure Purpose

data integration or data mining or data link* or data shar* or data process* or data curation or data harmoniz*

Infrastructure Type

data repositories or data hub or data warehouse or cyberinfrastructure or composite datasets or Biorepository or cyberinfrastructure or Biomedical informatics or Biomedical Research or Precision Medicine or Information Systems or perioperative care

Hospital Setting

hospital*

SECOND PHASE - IEEE Xplore

The second query includes additional keywords retrieved from the first phase articles: "Privacy and security" **AND** "Data processing" **AND** "Decision Support System". The selected terms embedded in the search command are shown in the highlighted areas.

Privacy and Security

security of data or data privacy

Data processing and management

(medical administrative data processing) or (database management system*) or (medical data or Big data) and (healthcare or health care or e-Health or patient care)

Decision Support Systems

decision support system*

A2 Targeted web-based search of known institutional IDRs

**Integrated Data Repository**	**References (numbered from main text)**
Stanford University Medical Center<br>**STRIDE**	[18,19]<br><br>Stanford University. Infrastructure Solutions | Research IT | Stanford Medicine [Internet]. 2018 [cited 2018 Mar 26]. Available from: http://med.stanford.edu/researchit/infrastructure.html
Vanderbilt University Medical center<br>**BioVU and SD**	[39,40]
University of Pennsylvania<br>**WRDS**	Wharton Research Data Services. Wharton Research Data Services | WRDS [Internet]. 2018 [cited 2018 Mar 27]. Available from: http://www.whartonwrds.com/<br><br>Cohen MW. BCCH inquiry about WRDS Healthcare Research Initiative (personal communication). 2018.
University of Michigan<br>**MPOG**	[267]<br><br>MPOG. MPOG – Multicenter Perioperative Outcomes Group [Internet]. 2017 [cited 2018 Apr 3]. Available from: https://mpog.org/
Boston University<br>**Clinical Data Warehouse**	Boston University. [Internet]. Boston University Medical Campus and Boston Medical Center. [cited 2018 Apr 12]. Available from: http://www.bumc.bu.edu/ohra/using-bmc-and-chc-data-for-research-purposes/<br><br>Rosen L. What is the Clinical Data Warehouse? http://www.bumc.bu.edu/crro/files/2010/01/Rosen-4-11-07.pdf
The Children's Hospital of Philadelphia<br>D3b<br>**BRP**	[41]<br><br>CHOP. About [Internet]. Children's Hospital of Philadelphia® Center for Data-Driven Discovery in Biomedicine. 2018 [cited 2018 Apr 17]. Available from: https://d3b.center/aboutd3b/history/
Veteran Health Administration<br>**VA HER**	[32]

A3 Additional references consulted during the synthesis stage

Additional information about the selected architectures for the comparative review analysis described in Table 1 of the main text. The references are shown as archived web-pages. Twenty out of the thirty-one selected architectures did not have detailed additional information to be found and are not listed in this table.

Institute	IDR	Archived reference and GitHub repos
The National Institutes of Health of Health Clinical Center	Biomedical Translational Research Information System (BTRIS)	Official BTRIS web page: http://archive.is/jbbnM<br><br>BTRIS presentation: http://archive.fo/sNDwt
Hanover Peter L. Reichertz Institute	Hanover Medical School Translational Research framework (HaMSTR)	Official Hannover Medical School Translational Research Framework (HaMSTR) web page: http://archive.is/KXmKI
Main partner: Cincinnati Children's Hospital Medical Center	Maternal and Infant Data Hub (MIDH)	News about MIDH at the Cincinnati Medical Hospital: http://archive.is/7TUZY
University of Kansas Medical Centre	Healthcare Enterprise Repository for Ontological Narration (HERON)	Wiki page of HERON: https://archive.is/uMSR6<br><br>HERON training material: http://archive.fo/GwtWd
Stanford University Medical Center	Stanford Translational Research Integrated Database Environment (STRIDE), STAnford Research Repository (STARR)	Resources at Stanford University Medical Center: http://archive.is/9wgGh<br><br>PDF - Description of IT managed resources: http://archive.is/N3AjH
The Georges Pompidou University	HGP CDW platform	i2b2 Clinical Data Warehouse at the Pompidou University Hospital in Paris (APHP - HEGP) - https://web.archive.org/web/20200423231835/ http://geneticalliance.org/sites/default/files/webinararchive/052214Avillach.pdf

Hospital (HEGP)		
Georges Pompidou, Cochin and Necker Hospitals	CAncer Research and PErsonalized Medicine (CARPEM)	Official CARPEM web page: http://archive.is/LFxdX
Learning Healthcare System (LHS) across South Carolina	Health Science South Carolina (HSSC) clinical data warehouse	i2b2 in the South Carolina Integrated Platform for Research - SCIPR: http://archive.is/5nUHz
Veterans Health Administration (VHA)	VA EHR (Veterans Administration Electronic Health Records)	Official VA web page: http://archive.is/oefaw<br><br>Health Affairs - Insights from Advanced Analytics at the Veterans Health Administration: http://archive.fo/boxkh
Coordinated by Medtronic Iberica SA	Models and Simulation Techniques for Discovering Diabetes Influence Factors (MOSAIC)	Official MOSAIC web page: http://archive.fo/B3NUN
Houston Methodist Hospital	Methodist Environment for Translational Enhancement and Outcomes Research (METEOR)	Official METEOR web page: https://archive.is/hRGAt<br><br>METEOR architecture: http://archive.fo/TX1cQ<br><br>METEOR data types: http://archive.fo/W50eV<br><br>Wiki page of METEOR: http://archive.fo/XgfvE
Vanderbilt University Medical Center	Synthetic Derivative (SD), BioVU	BioVU description at VUMC: http://archive.fo/8JwDh<br><br>BioVU and Synthetic Derivative: http://archive.fo/S8rjm<br><br>Synthetic Derivative: http://archive.fo/6INVt
University of Pavia and Fondazione S. Maugeri	onco-i2b2	onco-i2b2 architecture: http://archive.fo/YLxjz

The Children's Hospital of Philadelphia	Biorepository Portal (BRP)	The BRP toolkit official web page: http://archive.fo/dejFE<br><br>chop-dbhi, biorepo-portal, (2019), https://github.com/chop-dbhi/biorepo-portal<br><br>chop-dbhi, ehb-service, (2019), https://github.com/chop-dbhi/ehb-service<br><br>chop-dbhi, ehb-datasources, (2019), https://github.com/chop-dbhi/ehb-datasources
University of San Paulo	BioBankWarden (BBW)	Biobank Warden project web page: http://archive.fo/TZq75
Main partner: University of Utah	Federated Utah Research and Translational Health electronic Repository (FURTHeR), OpenFurther	openfurther, further-open-doc, (2015), https://github.com/openfurther/further-open-doc
@neurIST European Project	@neurIST platform	Official web-page - http://archive.is/MHkEc<br><br>NeurIST workshop - https://slideplayer.com/slide/4925632/

A4 Selected articles (n=255) summary statistics

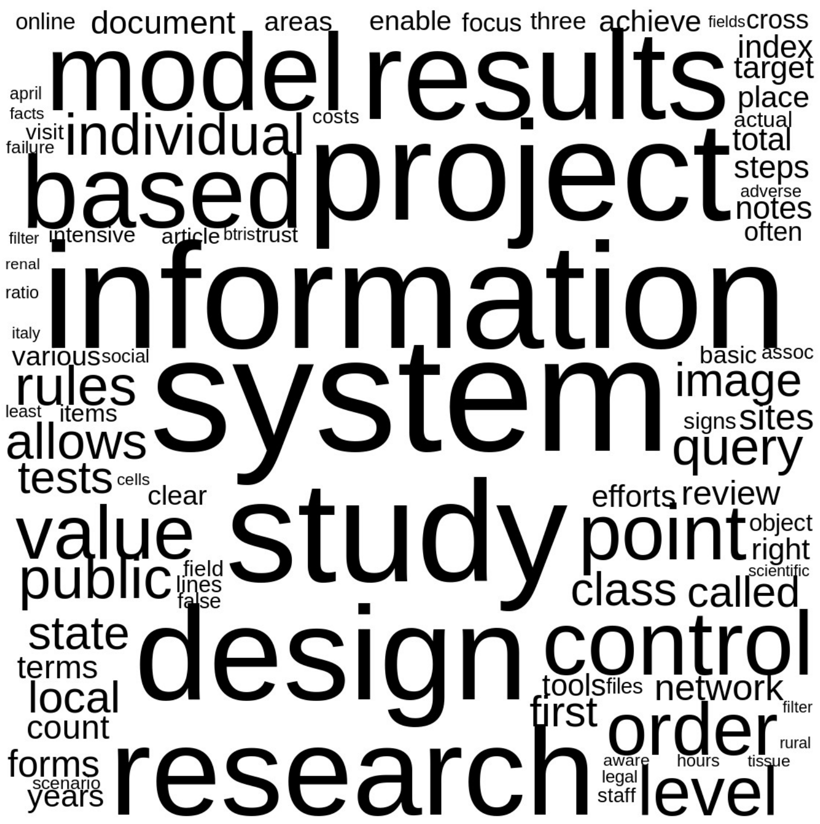

Figure A4.1 - Word cloud of article full text content.

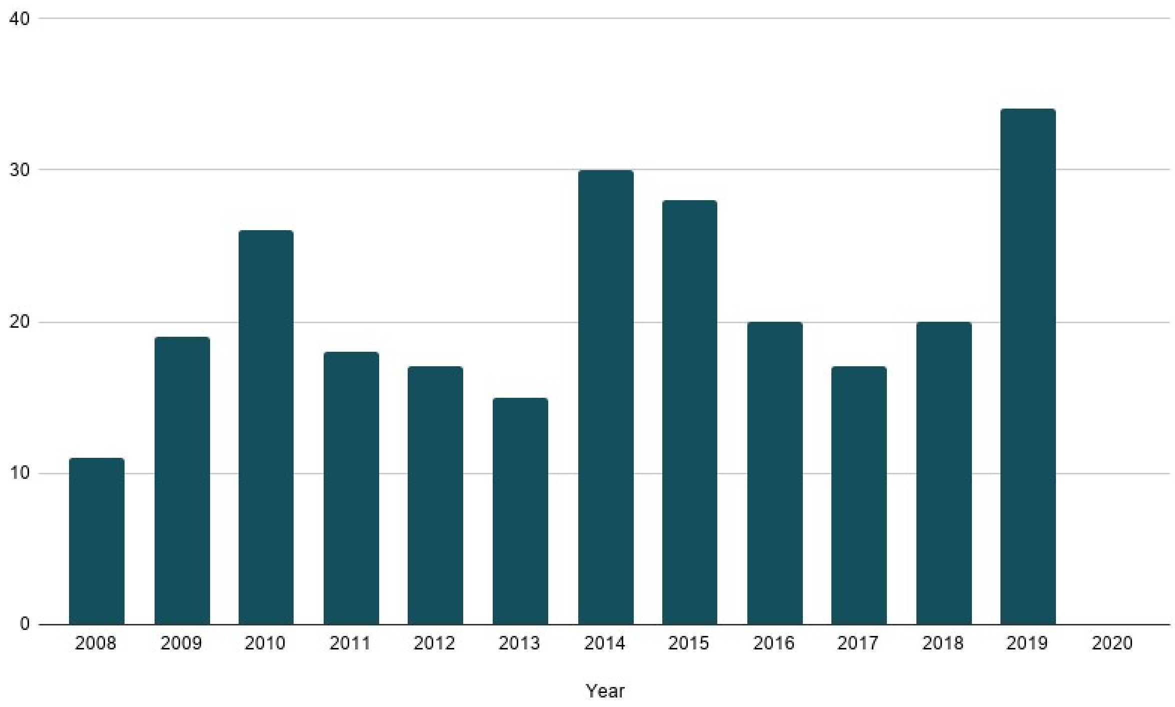

Figure A4.2 - Number of publications per year in the selected date range of 2008 - 2020

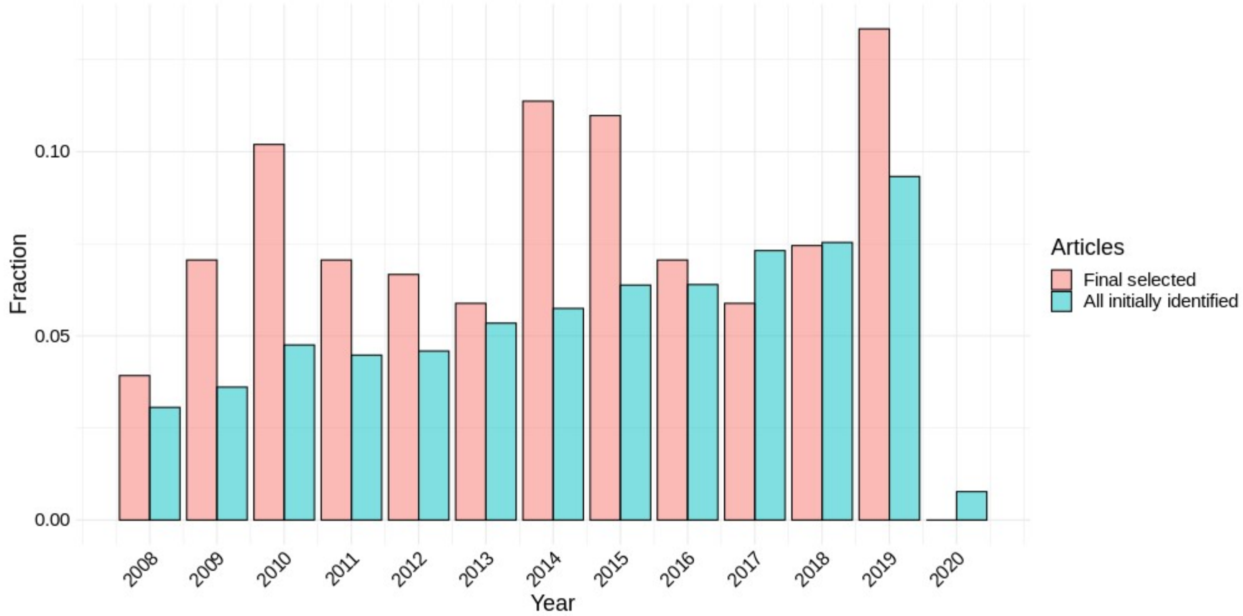

Figure A4.3 – Comparison of publication years for all initially identified articles (n=7,259) and the final set of selected articles (n=255) in the year range 2008 – 2020, shown as fraction of the total.

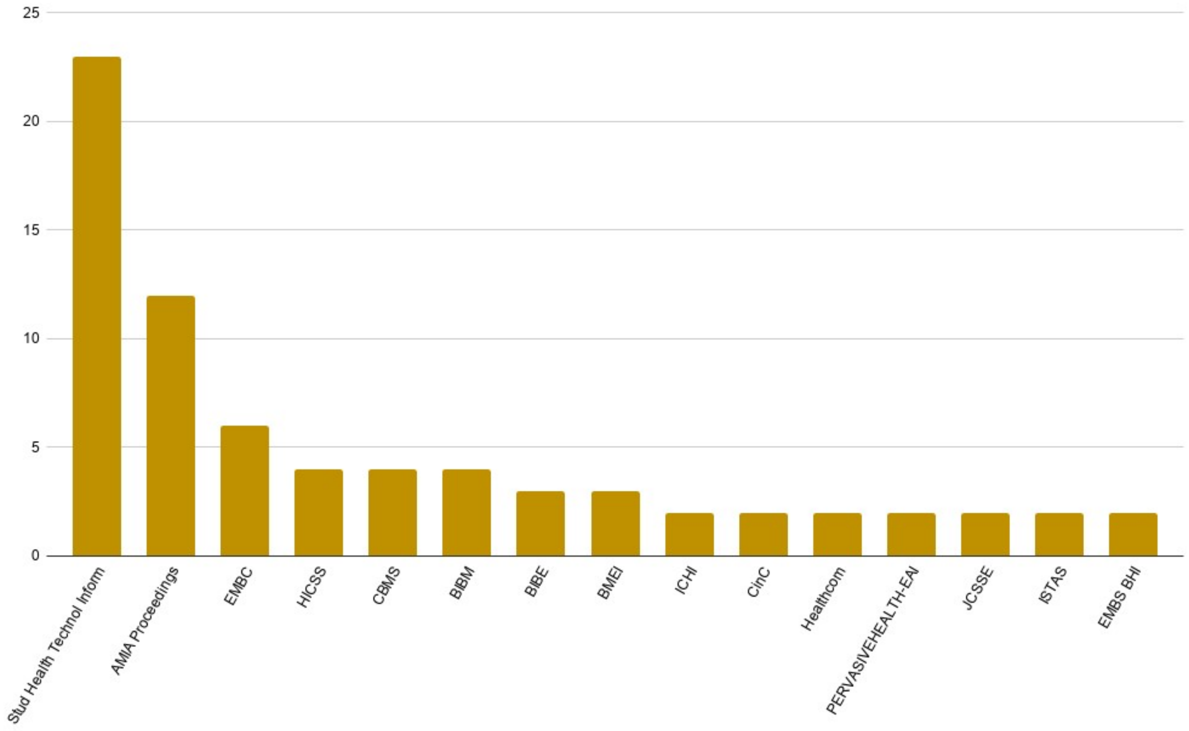

Figure A4.4 - Most frequent conference proceedings in which IDR papers are published: The Breakdown of conferences in "Studies in Health Technology and Informatics" (Stud Health Tech Info) and "American Medical Informatics Association Proceedings" (AMIA Proceedings) are as follows. Stud Health Tech Info: Word Congress of Medical and Health Informatics (n=9), Medical Informatics Europe (n=4), European Federation of Medical Informatics (n=3), eHealth (n=2), Informatics for Health (n=1), International Conference on Informatics, Management, and Technology in Healthcare (n=1), Information Technology and Communications in Health (n=1), Patient Safety Through Intelligent Procedures in Medication (n=1), pHealth, International Conference on Wearable Micro and Nano Technologies for Personalized Health (n=1); AMIA Proceedings: AMIA Annual Symposium Proceedings (n=10), AMIA Joint Summits on Translational Science Proceedings (n=2). ***Conferences abbreviations:*** AMIA - American Medical Informatics Association Proceedings; EMBC - International Conference in Engineering in Medicine and Biology Society; HICSS - Hawaii International Conference on System Sciences; CBMS - International Symposium on Computer-Based Medical Systems; BIBE - International Conference on BioInformatics and BioEngineering, BIBM - International Conference on Bioinformatics and Biomedicine, BMEI - International Conference on Biomedical Engineering and Informatics; ICHI - International Conference on Healthcare Informatics; CinC - Computing in Cardiology; Healthcom - International Conference on e-Health Networking, Applications and Services; PERVASIVEHEALTH-EAI - International Conference on Pervasive Computing Technologies for Healthcare; JCSSE - International Joint Conference on Computer Science and Software Engineering; ISTAS - International Symposium on Technology and Society; EMBS BHI - EMBS International Conference on Biomedical & Health Informatics

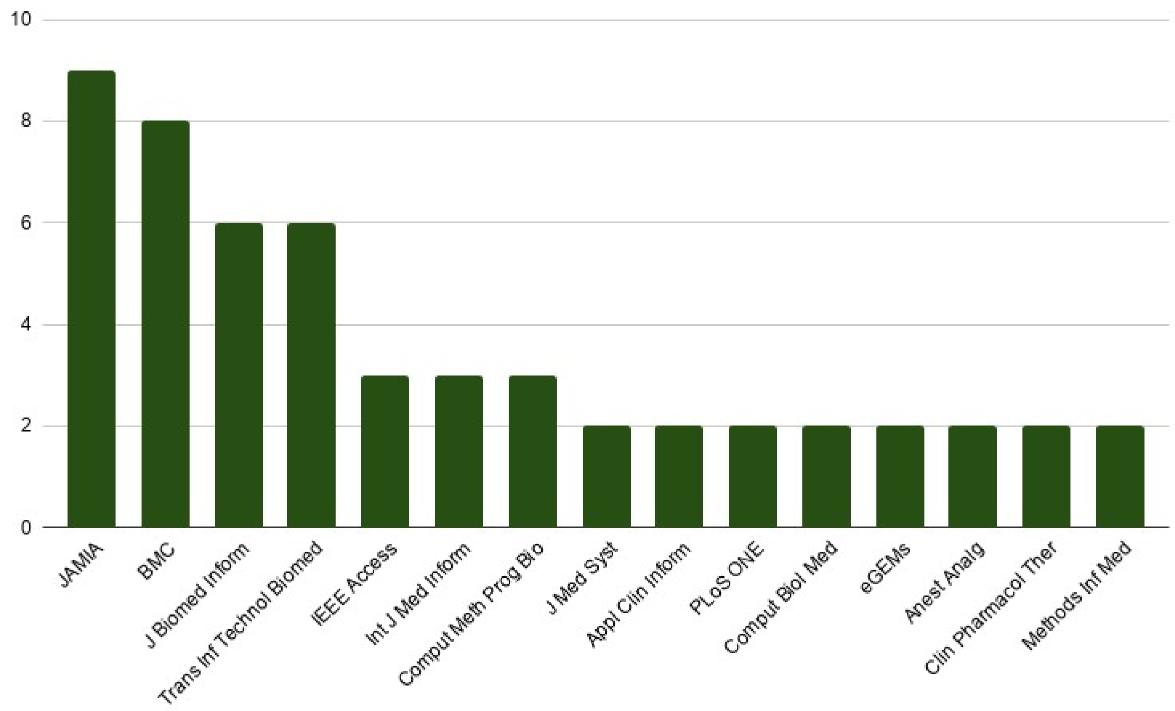

Figure A4.5 - Most frequent journals in which IDR papers are published. The list of BioMed Central (BMC) journals includes: BMC Bioinformatics (n=3), BMC Medical Informatics and Decision Making (n=2), BMC Medical Ethics (n=1), BMC Systems Biology (n=1), and BMC Genomics (n=1). ***Journal abbreviations:*** JAMIA - Journal of the American Medical Informatics Association; BMC - BioMed Central; ; J Biomed Inform - Journal of Biomedical Informatics; Trans Inf Technol Biomed - Transactions on Information Technology in Biomedicine; IEEE access – Institute of Electrical Electronics Engineers access; Int J Med Inform - International Journal of medical informatics; Comput Meth Prog Bio - Computer Methods and Programs in Biomedicine; J Med Syst - Journal of Medical Systems; Appl Clin Inform - Applied Clinical Informatics; Comput Biol Med - Computers in Biology and Medicine; Clin Pharmacol Ther - Clinical Pharmacology & Therapeutics; Anest Analg - Anesthesia and analgesia; Methods Inf Med - Methods of information in medicine

A5 Citations overlap of the main IDR articles

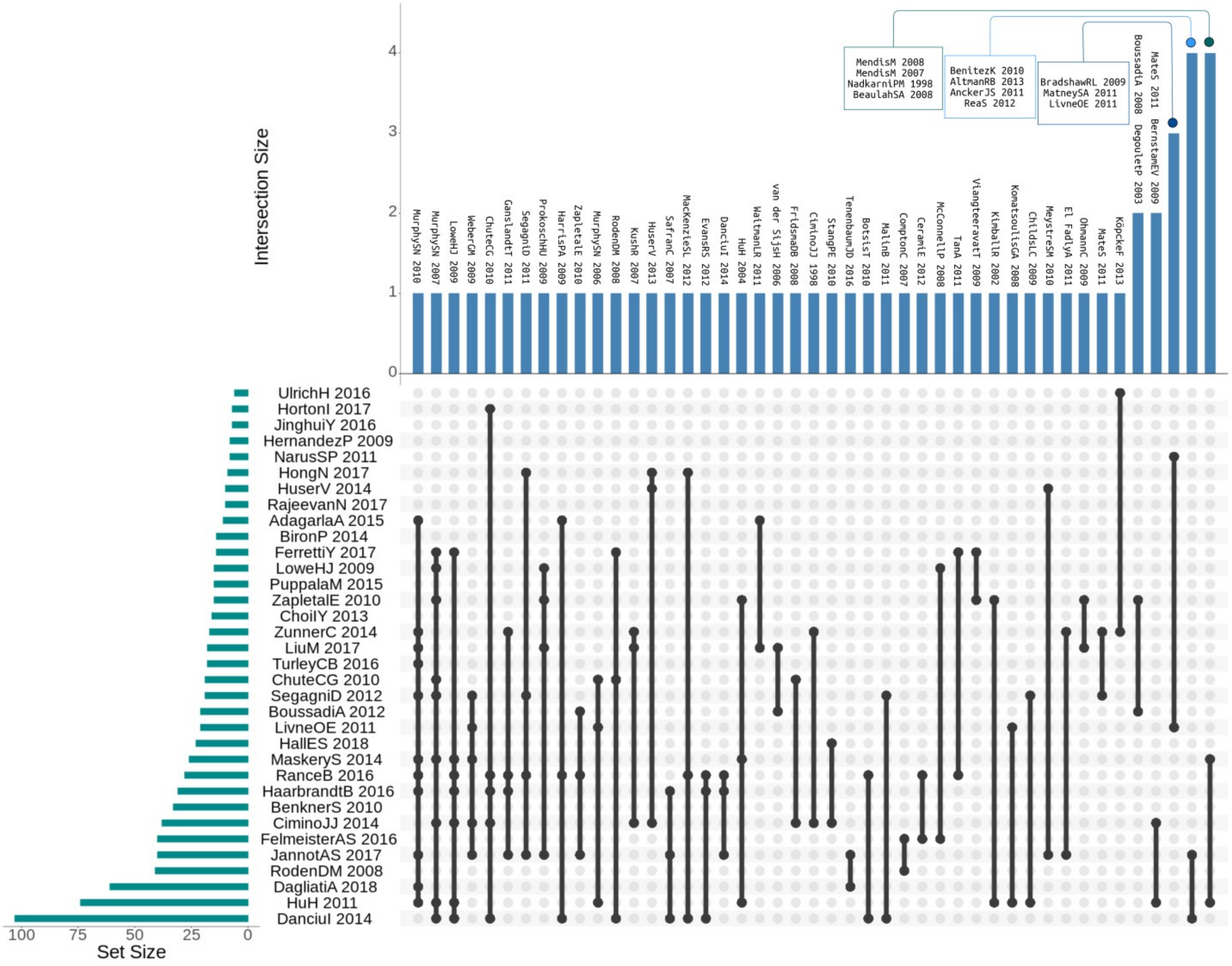

Figure A5.1 – UpSet plot of common citations across the selected 34 articles – The plot shows the number of citations (Set size) and the number of overlapping references (Intersection size) for each of the 34 articles listed in Table 1. References with no overlap are not displayed. The labels on the top of the bars show the name of the reference in the intersection set. As an example, the first column shows that one reference is found in 11 articles, while the last column shows that a group of four references can be found in common in two articles. More details in Lex *et al.*, 2014.

Table A5.2 – Frequency of the most cited articles among the 34 selected articles from Table 1. Reference numbers refer to the full citation in the main text.

Reference	Frequency
Murphy, S. N., *et al.* (2010) [277]	11
Murphy, S. N., *et al.* (2007)	9
Lowe, H. J., *et al.* (2009) [18]	8
Chute, C. G., *et al.* (2010) [36]	5
Weber, G. M., *et al.* (2009)	5
Ganslandt, T., *et al.* (2011)	4
Prokosch, H. U., & Ganslandt, T. (2009)	4
Segagni, D., *et al.* (2011) [43]<br>Segagni, D., *et al.* (2011) [271]	4
